# Response Comment on “Challenges and the Path Forward on Malaria Elimination Intervention: A Systematic Review”

**Published:** 2020-05

**Authors:** Khandan SHAHANDEH, Hamid Reza BASSERI

**Affiliations:** 1. Deputy of Research and Technology, Tehran University of Medical Sciences, Tehran, Iran; 2. Department of Medical Entomology and Vector Control, School of Public Health, Tehran University of Medical Sciences, Tehran, Iran

## Dear Editor-in-Chief

Thank you for providing us this opportunity to respond to the issues raised in the letter provided by Ahad Heydari and Saeed Fallah Ali Abadi and to explain our reasons in relation to those concerns. We would like also thanks the authors for their interest in our article and the time to provide their comments.

In their letter, the authors mentioned, as each systematic review is better to have protocol registration in order to prevent duplicating same research while conducting and finishing the systematic reviews. Indeed, we conducted the steps in the systematic review process as explained by several authors (1–7). The processes consisted of framing questions for the review; identifying relevant literature; assessing the quality of the literature; summarizing the evidence and interpreting the finding. In addition, according to guidelines for systematic reviews, revised 2017(8, 9), we develop our review protocol.

Our method for conducting the systematic review, describe the following: Step1. Framing questions for the review: Framing the question, by defining the PICO (Population, Intervention, Comparison, and Outcome) elements. Step2. Identifying relevant literature: generating the list of relevant citations to address our review questions, selecting relevant databases to search, and selecting general databases like MEDLINE which is available freely via PubMed, Web of Science and PubMed via their “Related Articles” function. Search strategy, search terms and the resources to be searched based on our review questions. Thus, after formulate our review questions and identify our key concepts, we develop search terms (free-text terms) and controlled vocabulary /MeSH terms for example health intervention, Malaria elimination, challenges, as well as combinations of these terms. List who conducted the search (e.g., independent librarian, librarian associated with author’s institution). Searching for study designs and reference lists and gray literature (Grey literature obtained after Google scholar, these databases). Procedures for identification and collection of articles.

List databases and other information sources used to identify relevant studies (e.g., hand-searching reference lists and tables of contents, contacting content experts). Include dates of coverage of the search as it is mentioned PRISMA Item #7 (3, 10). State the process for selecting studies (i.e., screening, eligibility, criteria for inclusion in systematic review) it is mentioned as PRISMA Item #9 (3, 10).

Study characteristics (PICO) and report characteristics (such as years considered, language, and publication status) used as criteria for eligibility. The criteria for study inclusion included the English language, interventional or observational studies, and analytic assessment of a health program. .Excluded records according to eligibility criteria such as letter to editor, dated before 1995, the study which duplicates the results of a previous study and editorial.

To screen citations for relevant to our review question, selecting relevant studies by defining the selection criteria in terms of the populations, interventions, outcome, and study design, to avoid bias in the selection process. Only studies that meet all of the inclusion criteria were included.

Screening the citations: Title and abstract screening and full-text screening conducted independently by two reviewers. For each study finally selected for inclusion, data extraction form (the form tailored to our research question to ensure we obtain all relevant information from each of the included study, which has been completed. Specify study characteristics (e.g., PICOS) and report characteristics (e.g., years considered, language, publication status) used as criteria for eligibility, giving rationale (PRISMA Item #6). Present our electronic search strategy for databases, and report the search terms (PRISMA Item #8). Based on the Cochrane Consumers and Communication Review Group's data extraction template, the data extraction form developed and piloted (PRISMA Item #10). List and define all variables for which data were sought (e.g., PICO, funding sources) and any assumptions and simplifications was made. (PRISMA Item #11). Describe method for assessing risk of bias of individual studies (including specification of whether this was done at the study or outcome level and how this information is to be used in any data synthesis such as strength of evidence assessments (PRISMA Item #12). The principal summary measures (e.g., risk ratio, difference in means) was stated (PRISMA Item #13). The method of handling data and combining results of studies was describe, if done, including measures of consistency (e.g., I2) for each meta-analysis (PRISMA Item #14). Any assessment of risk of bias that may affect the cumulative evidence (e.g., publication bias, selective reporting within studies) was Specified (PRISMA Item #15). In step 3, the quality of studies were assess by checking appropriateness of study design to the research objective, sample representativeness, risk of bias and other issues related to study quality. For developing our quality assessment checklist, key biases include selection bias, performance bias, measurement bias and attrition biases were considered by using the Cochrane risk of bias tool (11). Extracting information regarding study design, subjects, program and the level of intervention was performed.

In step 4, summarizing the evidence, the data from the studies narratively synthesis were carried out. Using PRISMA statement for presenting results. Finally, in step 5, interpreting the finding, heterogeneity, risk of bias (risk of publication bias and related biases) were explored and also assigning levels of evidence to recommendation was considered.

The authors pointed out to present search strategy for at least one database. In this regard, there is evidence that limiting the search to only a few databases tends to bias the review. As Aromataris & Riitano, 2014 stated that “, A systematic review requires a comprehensive search of multiple databases, using the same search strategy for each database (12). It is important that the protocol clearly outlines the planned search strategy; it ensures the search is undertaken in exactly the same way each time, and also allows the search to be replicated by other researchers in the future with the same results (12, 13). Grey literature is the term given to unpublished studies, theses, conference proceedings, presentations, government documents, or any other relevant documents that are not published in journals and will not appear in a database search (13, 14). The inclusion of grey literature helps to reduce publication bias. The notion that studies with limited, negative, or neutral outcomes are less likely to be published (13, 15). Besides, as many studies published are not included in general databases and journals, we searched other resources like gray literature in our review protocol.
